# Determinants of hospital nurse intention to remain employed: broadening our understanding

**DOI:** 10.1111/j.1365-2648.2009.05190.x

**Published:** 2010-01

**Authors:** Ann E Tourangeau, Greta Cummings, Lisa A Cranley, Era Mae Ferron, Sarah Harvey

**Affiliations:** Lawrence S. Bloomberg Faculty of Nursing, University of TorontoOntario, Canada; Faculty of Nursing, University of AlbertaEdmonton, Canada; Faculty of Nursing, University of AlbertaEdmonton, Canada; Lawrence S. Bloomberg Faculty of Nursing, University of TorontoOntario, Canada; Lawrence S. Bloomberg Faculty of Nursing, University of TorontoOntario, Canada

**Keywords:** employment, focus groups, hospital nurses, nursing workforce, theory development, work environments

## Abstract

**Title:**

**Determinants of hospital nurse intention to remain employed: broadening ourunderstanding.**

**Aim:**

This paper is a report of a study to identify nurse reported determinants of intention to remain employed and to develop a model explaining determinants of hospital nurse intention to remain employed.

**Background:**

A worsening shortage of nurses globally suggests that efforts must be made to promote retention of nurses. However, effective retention promotion strategies depend on understanding the factors influencing nurse retention.

**Methods:**

A descriptive study using focus group methodology was implemented. Thirteen focus groups including 78 nurses were carried out in two Canadian provinces in 2007. Thematic analysis strategies were incorporated to analyse the data.

**Findings:**

Eight thematic categories reflecting factors nurses described as influencing their intentions to remain employed emerged from focus groups: (1) relationships with co-workers, (2) condition of the work environment, (3) relationship with and support from one’s manager, (4) work rewards, (5) organizational support and practices, (6) physical and psychological responses to work, (7) patient relationships and other job content, and (8) external factors. A model of determinants of hospital nurse intention to remain employed is hypothesized.

**Conclusion:**

Findings were both similar to and different from previous research. The overriding concept of job satisfaction was not found. Rather, nurse assessments of satisfaction within eight thematic categories were found to influence intentions to remain employed. Further testing of the hypothesized model is required to determine its global utility. Understanding determinants of intention to remain employed can lead to development of strategies that strengthen nurse retention. Incorporation of this knowledge in nurse education programmes is essential.

## Introduction

The shortage of Registered Nurses (RNs) is a worldwide concern. In Canada, a shortfall of 113,000 RNs is expected by the year 2016 (Canadian Nurses Association 2002). Without an adequate supply of RNs, RN roles will be filled by healthcare providers with less knowledge and skill. There is clear evidence of the impact of less prepared staff delivering nursing care on important patient and organizational outcomes. For example, mortality rates for hospitalized patients are statistically significantly higher when lower proportions of RNs provide care (Aiken *et al.* 2002, Needleman *et al.* 2002, Tourangeau *et al.* 2002, 2007, Estabrooks *et al.* 2005).

Nurse turnover is a statistically significant contributing factor to the shortage of nurses (Mueller & Price 1990) and has been linked to decreased productivity, poor care quality, heavier workloads for remaining staff, decreased morale, increased potential for injuries, and further turnover (Health Canada 2007). Unfortunately, turnover rates of new nurses are high. Bowles and Candela (2005) reported that 30% of new nurses left their jobs in the first year and 57% resigned in their second year. There is no doubt of the importance of promotion of nurse retention as an approach to ensuring an adequate supply of RNs (Health Canada 2007). A number of strategies have been initiated to increase the supply of nurses including increasing nursing education seats and discouraging nurses from early retirement but such strategies may be ineffective if not developed on sound understanding of factors that influence nurse intent to remain in or leave employment. This paper focuses on new research exploring factors influencing nurse retention.

## Background

### Theoretical perspectives

There is a direct link between intention to remain employed and retention of employees. Intent to remain employed is a strong statistically significant predictor through which other factors operate to affect retention behaviour (Price & Mueller 1981, Hinshaw *et al.* 1987, Tett & Meyer 1993, Hom & Griffeth 1995, Irvine & Evans 1995, Taunton *et al.* 1997, Griffeth *et al.* 2000, Price 2001, van Breukelen *et al.* 2004). Although retention theories differ in their complexity and inclusion of determinant variables, their commonality lies in their propositions of a multistage process that is attitudinal, decisional and behavioural. The Theory of Reasoned Action suggests that attitudes affect decisions and ultimately behaviour (Fishbein & Ajzen 1975, Ajzen & Fishbein 1980). Accordingly, nurse attitudes about work affect their decisions (intentions) to remain employed and ultimately their actions (retention or termination).

Previous researchers have had limited success in explaining determinants of nurse intent to remain employed. When testing hypothesized models of determinants of nurse intention to remain employed, between 22% and 52% of the variation in nurse intention to remain employed was explained (Taunton *et al.* 1997, Boyle *et al.* 1999, Sourdif 2004, Tourangeau & Cranley 2006). Given the magnitude of unexplained variance, there are clearly unknown factors influencing nurse intention to remain employed.

Several theories explaining determinants of nurse intention to remain employed have been hypothesized and tested. Consistent among theoretical perspectives is the impact that job satisfaction has on nurse intention to remain employed. Job satisfaction as a construct has been reported to be one of the most important factors influencing nurses’ decisions to remain in or leave their jobs. Boyle *et al.* (1999) hypothesized that perceptions of nurse-manager’s leadership style, nurse job satisfaction, job stress, work autonomy and group cohesion directly influenced nurse intent to remain employed. They reported that job satisfaction had the strongest influence on intent to remain employed but also that nurse-manager leadership style directly influenced nurse intent to stay. Job stress, autonomy and group cohesion indirectly influenced nurse intent to remain employed through job satisfaction. Sourdif (2004) hypothesized that nurse intent to stay was influenced by satisfaction at work, satisfaction with administration, organizational commitment, and work group cohesion. Sourdif found that work satisfaction and satisfaction with administration were the most significant predictors of intent to remain and explained 25·5% of variance in intent to remain. Tourangeau and Cranley (2006) hypothesized that job satisfaction, organizational commitment, work group cohesion and collaboration, manager ability and support, burnout, and nurse characteristics directly influenced nurse intent to remain employed. They found that age, organizational commitment, job satisfaction and work group cohesion were significant predictors of intent to remain. They found no evidence of a direct relationship between either manager ability and support or burnout with intention to remain employed. They and other researchers found that manager ability and support influenced nurse intent to remain employed indirectly through job satisfaction (Shader *et al.* 2001, Larrabee *et al.* 2003).

Based on a review of the theoretical and research literature, six general categories of determinants of nurse intention to remain employed have been reported: job satisfaction, organizational commitment, manager ability and support, work group cohesion, job stress and burnout, and nurse characteristics. Below, findings related to the impact of each of these categories of determinants are briefly highlighted.

### Job satisfaction

There has been an accumulation of research establishing job satisfaction as one of the most important predictors of nurse intention to remain employed (Mobley *et al.* 1978, Hinshaw *et al.* 1987, Tett & Meyer 1993, Tai *et al.* 1998, Boyle *et al.* 1999, Shader *et al.* 2001, Yin & Yang 2002, Larrabee *et al.* 2003, Sourdif 2004, Lu *et al.* 2005, Hayes *et al.* 2006, Tourangeau & Cranley 2006, Gregory *et al.* 2007, Lacey *et al.* 2007, Beecroft *et al.* 2008). This relationship has been found internationally. A major challenge across studies was how job satisfaction has been conceptualized and measured. In some studies, job satisfaction was conceptualized as a single summary construct while in others multiple components of job satisfaction reflecting various aspects of work have been considered.

### Organizational commitment

There is some evidence that organizational commitment, defined as a psychological state binding an employee to the organization (Allen & Meyer 1990), is a greater predictor of intention to remain than is job satisfaction. In a meta-analysis of published studies in management, psychology, and sociology literatures, Griffeth *et al.* (2000) concluded that organizational commitment predicted turnover better than did job satisfaction. Similarly, Ingersoll *et al.* (2002) found that organizational commitment was a stronger predictor of RN intent to stay than was job satisfaction.

### Manager ability and support

Boyle *et al.* (1999) examined the effects of nurse manager characteristics of power, influence, and leadership style on critical care nurse intention to remain employed. They found that nurses with higher intention to stay expressed higher influence of the manager over how staff did their jobs and solved problems, higher perceived ability of the manager to control others through the use of reward and punishment, higher opportunities for promotion, and lower availability of alternative jobs in the community. Taunton *et al.* (1997) reported similar findings. Tourangeau and Cranley (2006) found no direct relationship between manager support and nurse intention to remain employed but hypothesized that manager support indirectly affects intent to remain employed, mediated through job satisfaction.

### Work group relationships and cohesion

Healthy and supportive work relationships have been shown to be related to higher nurse intention to remain employed (Hinshaw *et al.* 1987, Shader *et al.* 2001, Sourdif 2004, Tourangeau & Cranley 2006). Interestingly, Beecroft *et al.* (2008) found that the more social support strategies that new graduate nurses accessed, the higher was the risk of these new nurses leaving their jobs.

### Stress and burnout

Work-related stress and burnout have been found to be statistically significant predictors of intention to turnover in Canadian nurses (Zeytinoglu *et al.* 2006, 2007). In a Taiwanese meta-analysis of studies, Yin and Yang (2002) found that stress associated with high workload was the second most frequent reason nurses left their jobs. Hinshaw *et al.* (1987) reported that job stress was a predictor of anticipated turnover for nurses, which operates through organizational and professional job satisfaction. Similarly, Boyle *et al.* (1999) reported that job stress was indirectly related to intention to turnover through job satisfaction. Tourangeau and Cranley (2006) did not find a direct relationship between nurse burnout and intent to remain employed but suggested that nurse burnout indirectly effects intention to remain employed through job satisfaction.

### Nurse characteristics

Nurse characteristics found to be associated with intention to remain employed include years of experience (Price & Mueller 1981, Irvine & Evans 1995), educational preparation (Tai *et al.* 1998, Yin & Yang 2002, Tourangeau & Cranley 2006, Coomber & Barriball 2007), and age (Irvine & Evans 1995, Tai *et al.* 1998, Shader *et al.* 2001, Tourangeau & Cranley 2006). Nurse age in years may provide limited explanatory power. Instead, Tourangeau and Cranley (2006) hypothesized that the relationship between age and intention to remain employed is a function of different attitudes, values, goals and expectations held by generational affiliation.

## The study

### Aims

The aims of the study were to identify nurse reported determinants of intention to remain employed and develop a model explaining determinants of hospital nurse intention to remain employed.

### Design

A descriptive study using focus group methodology was carried out.

### Participants

There are four generations of nurses in the workforce (Duchscher & Cowin 2004), with each having differing life and work values, goals and attitudes (Hu *et al.* 2004). Given differences across generational cohorts, it is reasonable to suspect that factors influencing their intention to remain employed may also differ in nature and strength (Zemke *et al.* 2000, McNeese-Smith & Crook 2003, Wilson-Keates *et al.* 2008). In this study, we focused on nurses in the three youngest generations (Duchscher & Cowin 2004): Baby Boomers (born between 1946 and 1964), Generation X (born between 1965 and 1979) and Generation Y or Millenials (born after 1979). Nurses in the eldest generation, the Silent Generation who were born in or before 1945, were not included in this study as they will all reach the normal age of retirement in 2010.

Purposive sampling strategies were incorporated to enlist focus group participants. The inclusion criteria were:

- RN working on medical, surgical, or critical care hospital areas.
- Born 1946 or later.
- Able to participate for a 60–90 minute period in designated room on hospital campus.
- Able to speak and read English.
- Able to provide informed and written consent.

Nurses from six hospitals were invited to participate in one of 13 focus groups in 2007. Hospitals were selected based on nurse leadership interest in the study. In each of two Canadian Provinces, Alberta and Ontario, nurses from one large teaching hospital, one large community hospital, and one rural or remote hospital were invited to participate.

Although more than 125 nurses expressed a desire to participate in focus groups, 78 RNs participated in one of 13 focus groups, with an average of six participants per group (range 3–16). The remaining nurses were unable to participate at the scheduled focus group dates and times. Table 1 shows the number of participants in each province by generational affiliation.

**Table 1:** Focus group participant numbers by province and generational affiliation

Province	Baby Boomer (born 1946–1964)	Generation X (born 1965–1979)	Generation Y (born 1980 onward)	Total
Ontario	15	15	3	33
Alberta	21	14	10	45
Total	36	29	13	78

Nurses were invited to participate through two mechanisms, i.e. email invitations and invitation flyers posted in staff areas. Both invitations included introductory study information; participant inclusion criteria; date, time and location of scheduled focus groups at their hospital; and a free telephone number to contact the research team. A light meal was provided at each focus group and participants were offered a gift card valued at 30 Canadian dollars in appreciation for participating.

### Data collection

Each focus group was led by two moderators consisting of a researcher and a research assistant. One moderator led the group and the other attended to participant needs, functioning and location of digital recorders, and ensuring a comfortable environment. Focus groups were digitally recorded, transcribed verbatim, and transcripts were given to the research team for analysis.

A semi-structured question guide was prepared that included one main question and several probing questions. Focus groups were opened with one question reflecting the study research question: What circumstances in your work or life influence your decision to remain in or leave employment in your job at this hospital? Probing questions were developed based on current knowledge of determinants of intention to remain employed from the literature but were never required. Moderators sought clarification intermittently.

### Ethical considerations

Approval for the study was obtained from the appropriate ethics review panels.

### Data analysis

Thematic analysis strategies were used. Analysis began with the first focus group and was conducted concurrently throughout data collection. Each transcript was carefully read and re-read to understand the content. An initial set of broad codes was assigned to the text based on recurring patterns and themes in the data and to identify core meanings (Miles & Huberman 1994). Codes were grouped into categories and we sought a balance that acknowledged the interplay between individual participants and the group as levels of analysis (Morgan 1997). Particular attention was given to words and phrases that participants used to describe factors influencing their intent to remain employed. Further interpretive reading of the transcripts and a more focused coding was used to refine the categories and emerging themes (Krueger 1998). Data were continuously compared and contrasted across groups and subsequent focus group data were analysed and compared to earlier groups (Krueger & Casey 2000).

Four criteria outlined by Lincoln and Guba (1985) were used to establish trustworthiness of data. Credibility was established through use of verbatim quotes to illustrate findings and through member checking. We presented preliminary findings to some participants, requesting their views on accuracy of interpretation. Transferability of findings was supported through descriptions of the time and context in which these data were found. These allow readers to make decisions about transferability. Both dependability and confirmability were strengthened through an audit trail (e.g., transcripts, history of theme development).

## Findings

Focus group data yielded eight thematic categories of factors influencing hospital nurse intention to remain employed: relationships with co-workers, condition of the work environment, relationship with and support from one’s manager, work rewards, organizational support and practices, physical and psychological responses to work, patient relationships and job content, and external factors. Each thematic category is described below.

### Relationships with co-workers

In all focus groups, participants discussed the importance of their relationships with co-workers. Some indicated that the nature and quality of these relationships was the most important reason that they stayed employed, and others identified that negative or unsatisfying co-worker relationships were a strong impetus for leaving their jobs. Nurses described how having a sense of belonging with a peer group was an important reason to stay employed with that group. For example, one nurse described the importance of co-workers:

My colleagues – that’s the only thing that makes me want to stay here, because we work with a lot of very good people, they’re professional, and that’s the one driving force I think that keeps most of us here.

Nurses wanted to stay employed when they felt that their work group was stable and dependable. When they trusted and respected each other and other team members, particularly physicians, they described being more inclined to remain in their job. Having regular opportunities to socialize and celebrate with co-workers was seen as an important component of belonging to that team.

Some participants described being involved in or witnessing nurse-to-nurse or nurse-to-other co-worker situations of bullying or belittling. Those who discussed such situations all identified these as motivators to leave their jobs. One gave the following reason for considering leaving her job:

I’ve never been screamed at by a co-worker until this past year, I have never been disrespected the way I have since I’ve been working this position.

### Condition of the work environment

In all focus groups, participants discussed the work environment as an important factor influencing their intentions to remain employed. Central to these discussions was the perceived adequacy of both human and material resources for providing patient care. Nurses reported being more inclined to consider leaving their jobs when there were inadequate numbers of nursing and other staff, as well as when there were inadequately prepared staff to deliver nursing care. Participants sometimes described these situations as being unsafe workplace conditions that they desired to leave. They responded similarly when chronically faced with unavailability of needed material resources, primarily equipment and supplies. For example, one nurse stated:

A lot of the times when you go into get equipment and stuff it’s just not there. So you’re going into your medication cart... we have very quick turnover of patients so the drugs aren’t there, you’re constantly hunting for medication, the syringe bin is always empty, so then you’ve got to go and grab it. Even just looking for an IV pump, you’re constantly going in circles. You spend a lot of time just wasting time looking for stuff and getting equipment.

Interestingly, there was considerable discussion related to the condition of the physical work environment as influencing nurse intentions to remain employed. Nurses felt that supportive physical environments (clean air quality, clean environment, comfortable and safe furniture, and a private place for staff to relax) were motivators to remain employed.

### Relationship with and support from manager

In most focus groups, participant perceptions of the abilities, intentions and relationships with managers were discussed as influencing their intentions to remain employed. Many believed that their managers were not visible at work and that this was due to managers’ wide spans of control. Participants felt that their managers had direct influence on the work environment, work processes and work rewards such as praise and recognition, all of which influenced their intentions to remain employed. They described their expectations that managers be fair, respectful, supportive and have strong interpersonal skills. For example, one nurse gave the following reason for recently leaving her job:

… and that is why I left, was management. It was very incompetent, very unprofessional, very different type of nursing, and that’s why I cut my time short there because I had no support from her.

### Work rewards

Participants in every focus group identified several work rewards that influenced their intentions to remain employed. Commonly, salary was the work reward discussed. One nurse stated:

What will retain me here? Money.

Benefits were frequently seen as important rewards that encouraged nurses to remain employed, including pension benefits, parental leave, reasonably priced and safe parking, and access to fitness facilities. Having opportunities to schedule preferred vacation and holiday times were seen as powerful motivators to remain employed. Others believed that formal recognition for knowledge, experience and effort was strong incentive to remain employed.

Here, generational affiliation differences were obvious. Those with many years experience were most concerned about salary and pension benefits as important reasons to remain employed, while younger nurses were most concerned about parental leave and being able to take preferred vacations. Because of low seniority, many new nurses identified that they were unable to schedule vacations even for important life events such as their own weddings. Such situations were powerful incentives for new nurses to consider leaving their current employment. As one younger nurse said:

You don’t get your vacation, you don’t get your stats, you couldn’t get your educational days, you could not get things that were rightfully yours to ask for as per the collective agreement, you had to defend everything and fight for everything you wanted.

### Organizational support and practices

Nurses identified a variety of topics related to organizational support and practices as influencing their intentions to remain employed. They discussed the impact of organizational support for professional nursing practice, particularly related to access to funded internal and external educational opportunities as important reasons for staying employed. Also discussed was the importance of having adequate orientation and on-going formal support for newly hired nurses. One nurse described the positive impact of educational opportunities:

The positive things that keep you here and employed within this institution … that there’s always some educational offering that could offer personal growth and development and opportunity.

Having meaningful opportunities to give input on hospital committees was discussed as supportive of nurse retention. However, sometimes these opportunities were seen as insincere when little or no support was provided to facilitate nurse participation (e.g. arrangements for patients to be cared for by other nurses).

Participants also discussed organizational support for accessible child and elder care. Hospitals that offered child or elder care services were described as being more desirable places to work. Some nurses identified the impact of having real opportunities for flexible time off to manage family responsibilities (e.g. child and/or elder care) on their intention to remain. They identified organizations with agreements to facilitate flexible time off as better organizations in which to work. Some discussed how flexible organizations that supported changes in job status promoted retention (e.g. job-sharing, movement between full and part time as life situations changed). One nurse described why she would be more inclined to remain employed:

If there were positions that have flexible hours for your family needs – if you could find a job like that!

There was considerable discussion of the impact of age-appropriate scheduling practices. Overall, hospitals that demonstrated ‘sensible’ flexibility were described as being better places to work. Participants were supportive of age and life-stage appropriate scheduling to promote retention. In particular, they discussed the positive impact of organizational practices to modify schedules and workloads for older nurses. One said:

I wish something more could be done to retain older nurses, because for myself I’m comfortable at some things but other people may not be. It’s heavy, nursing is heavy, and you can’t say when you get to be, let’s say, 55, go to a lighter area. There aren’t any lighter areas!

### Physical and psychological responses to work

Participant perceptions of their responses to work were identified as factors influencing their intent to remain employed. Nurses who identified being very stressed or burned out indicated that they were planning to leave employment because of the negative impact of work on their health. Often they described working ‘too hard, too much and too often’ as being an important consideration for wanting to leave their jobs. This was related to their descriptions of being unable to find an acceptable balance between work and personal life. Some described how they frequently were called on their mobile phones as they walked out of the hospital, asking them to return to work on their scheduled time off. One described how feeling overwhelmed was influencing her plans to leave her current job:

So that’s one of the reasons that I could see leaving nursing... we’re overworked now, stress and burnout I think is going to lead to a lot of us leaving.

### Patient relationships and job content

Participants discussed how positive connections with patients and families promoted their intentions to stay employed. Many discussed the importance of being stimulated with new patient challenges as motivators to stay in their current jobs. For example, one stated:

…days where I don’t feel challenged …I feel, okay it’s time for me to move on, it’s time for me to learn something.

Nurses also described wanting manageable workloads. Some described overwhelming feelings of frustration and even failure when unable to provide adequate patient care because of workload pressures. These situations were described as being central to wanting to leave their jobs. Another nurse described what was making her think of leaving her job:

The days that I’m too rushed, and my patients are like half taken-care-of.

Patient flow issues were cited as sources of frustration. Routine exposure to lack of beds, overcrowding and over-census on units were discussed as incentive to seek a new job. These issues were seen as out of nurse control. They felt powerless to resolve patient flow issues. Nurses also identified requirements to engage in non-patient or non-therapeutic activities such as cleaning human waste or transporting patients outside to smoke as being incentives to leave one’s job. These situations were seen as further eroding time available to provide therapeutic nursing care.

### External factors

Several factors external to work employment were identified as influencing decisions to remain employed. Nurses described how favourable opportunities to work elsewhere influenced their intentions to remain employed. This impact was reported to be even stronger when they were being personally recruited by other organizations and offered incentives or bonuses to move to a new organization. For example, one described external incentives encouraging her to change jobs:

Incentives, pay incentives, any type of incentive to come and work in this hospital, sign-on bonuses.

**What is already known about this topic**

- There is a direct and positive relationship between intent to remain employed and retention of employees.
- Job satisfaction has been reported to be one of the most important factors influencing nurses’ decisions to remain in or to leave their jobs.
- There are unknown factors influencing nurse intentions to remain employed in acute care hospitals and across all generations in the workplace.

**What this paper adds**

- A model of determinants of hospital nurse intention to remain employed is advanced from eight thematic categories that emerged from the focus groups.
- Job satisfaction as a single thematic category was not found to be an overriding construct influencing nurses’ decisions to remain in or leave their jobs.
- Instead, nurse assessments of the adequacy of or satisfaction with each of the eight thematic categories influenced their intentions to remain in or leave employment.

**Implications for practice and/or policy**

- To promote retention of hospital nurses, focus should be placed on modifying work environment and organization characteristics rather than on modifying nurses and nurse behaviours.
- Further research is required to test and refine the hypothesized model of determinants of hospital nurse intention to remain employed and to explore differences in the strength of determinants across generations of hospital nurses in the workforce, as well as to test the model’s explanatory power internationally.

### Hypothesized model

**Figure 1:**
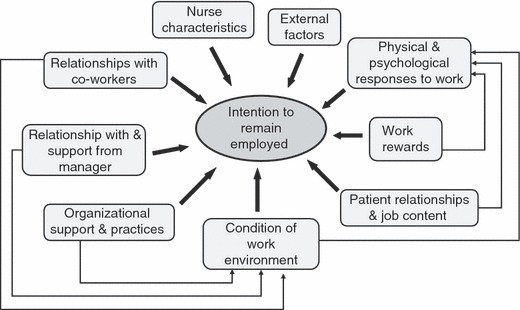
Determinants of Hospital Nurse Intention to Remain Employed Model. Note. Thick darker arrows leading directly from each thematic category (determinant) to intention to remain employed reflect direct effects or impacts of determinants on intention to remain employed. The narrower arrows around the perimeter of the model reflect the impact or effects of thematic categories (determinants) on other thematic categories which reflect additional indirect effects of determinants on intention to remain employed, mediated through other determinants.

Our hypothesized model of determinants of hospital nurse intention to remain employed, illustrated in Figure 1, was developed around the eight thematic categories that emerged from focus groups. Because of previous research, we have added one additional thematic category reflecting nurse characteristics. Although nurse characteristics are not modifiable, it is important to understand the impact they have on nurse intention to remain employed. Hypothesized relationships are based on focus group findings and on previous research findings that were highlighted in the background literature review. All determinants (thematic categories) are hypothesized to have a direct effect on intention to remain employed. Some determinants are hypothesized to influence other determinants. When one determinant is hypothesized to affect another, that determinant is proposed to have an additional indirect effect on intention to remain employed, mediated through another determinant.

## Discussion

Our findings appear to differ from previous findings that job satisfaction, as a composite construct, is the most consistent and possibly largest influencing factor on nurse intention to remain employed (Boyle *et al.* 1999, Sourdif 2004, Tourangeau & Cranley 2006). Participants never used the term job satisfaction as influencing their intention to remain employed. Instead, our findings suggest that nurse assessments of their satisfaction with their work and their hospitals in these eight thematic categories influence their intentions to remain. We suspect that this difference in findings may be an artifact of instruments used to measure job satisfaction. Job satisfaction measurement instruments commonly used in nursing research may measure nurse perceptions of satisfaction with some but not all influencing factors. These findings suggest that in research we need to ensure that the instruments we use measure the constructs being studied.

A second finding that differs from those of others relates to the impact of nurse assessments of organizational commitment on their intention to remain employed (Griffeth *et al.* 2000, Ingersoll *et al.* 2002). We suggest that it is nurse assessments of the adequacy of their organizations’ support and practices that influence their intentions to remain employed. These assessments might, in turn, be a precursor to summary nurse judgments of their organizational commitment. It is likely that nurse assessment of organizational commitment has acted as proxy for their assessments of the adequacy of or satisfaction with organizational support and practices.

Similar to findings of others (Hinshaw *et al.* 1987, Shader *et al.* 2001, Sourdif 2004, Tourangeau & Cranley 2006), we found that participants consistently reported the strong impact that co-worker relationships had on their employment intentions. We also found that nurse assessments of the adequacy of their relationships with and support from their managers influenced their intention to remain employed as did Boyle *et al.* (1999) and Taunton *et al.* (1997). Similarly, our participants reported that work-related responses such as stress and burnout influenced their intentions to remain employed. These findings support those of others (Hinshaw *et al.* 1987, Boyle *et al.* 1999, Yin & Yang 2002, Zeytinoglu *et al.* 2006).

### Study limitations

One study limitation is that we were unable to draw conclusions related to the strength of influencing factors across different generational nurse cohorts. Although we suspect that the strength of influencing factors varies depending on generational affiliation, further research is required to test this hypothesis. A survey of hospital nurses is currently underway to test and refine this proposed theory, as well as to examine differences in the strength of influencing factors across generations of nurses.

A second limitation reflects lack of knowledge about applicability of these findings across international hospital settings. Those aspects of work that promote or weaken nurse retention may differ in nature and strength across international boundaries, particularly in countries and cultures with different values and norms than those in this study. Such potential differences were not explored.

## Conclusion

If we are interested in promoting hospital nurse retention, we need to focus on modifying aspects of the eight thematic categories reflecting nurse satisfaction of their work and work environments rather than on modifying nurses. Nurse retention challenges and obstacles may be less about nurses and more about the organizations in which they work. Hospitals with issues of nurse retention are encouraged to examine how nurses rate each of these thematic categories with respect to their employment. Retention may be promoted by developing and implementing strategies that manage one or more of these determinants so that nurses are more satisfied with those aspects of work.

Our findings underscore the complexity of determinants of nurse intention to remain employed and related challenges associated with promoting nurse retention. Clearly, there is not one solution to promoting nurse retention because there is not one single cause or influencing factor. Several influencing factors relate to the importance of basic human relationships and remind us of the importance that human relationships have at work. In particular, relationships with co-workers and managers strongly influence nurses’ decisions about continuing in employment. Ignoring the importance of human relationships in promoting nurse retention may place all other retention promotion strategies at risk for failure. Strategies that focus on building respectful relationships at work may have tremendous capacity to promote nurse intention to remain employed. Knowledge and practice of relationship-building strategies should begin within both undergraduate and graduate educational programmes and should involve nurses and other members of the interdisciplinary health team.

## Author contributions

AT was responsible for the study conception and design. AT, GC and LC performed the data collection. AT, GC, LC, EF and SH performed the data analysis. AT, GC, LC, EF and SH were responsible for the drafting of the manuscript. AT, GC, LC, EF and SH made critical revisions to the paper for important intellectual content. AT, GC and LC obtained funding. AT provided administrative, technical or material support. AT supervised the study.

## References

[b1] Aiken LH, Clarke SP, Sloane DM, Sochalski J, Silber JH (2002). Hospital nurse staffing and patient mortality, nurse burnout, and job dissatisfaction. Journal of the American Medical Association.

[b2] Ajzen I, Fishbein M (1980). Understanding Attitudes and Predicting Social Behavior.

[b3] Allen NJ, Meyer JP (1990). Organizational socialization tactics: a longitudinal analysis of links to newcomers’ commitment and role orientation. Academy of Management Journal.

[b4] Beecroft PC, Dorey F, Wenten M (2008). Turnover intention in new graduate nurses: a multivariate analysis. Journal of Advanced Nursing.

[b5] Bowles CE, Candela LE (2005). First job experiences of recent RN graduates: improving the work environment. The Journal of Nursing Administration.

[b6] Boyle DK, Bott MJ, Hansen HE, Woods CQ, Taunton RL (1999). Managers’ leadership and critical care nurses’ intent to stay. American Journal of Critical Care.

[b7] van Breukelen S, van der Vlist R, Steensma H (2004). Voluntary employee turnover: combining variables from the ‘traditional’ turnover literature with the theory of planned behavior. Journal of Organizational Behavior.

[b8] Canadian Nurses Association (2002). http://www.cna-aiic.ca/CNA/issues/hhr/default_e.aspx.

[b9] Coomber B, Barriball KL (2007). Impact of job satisfaction components on intent to leave and turnover for hospital-based nurses: a review of the research literature. International Journal of Nursing Studies.

[b10] Duchscher JE, Cowin L (2004). Multigenerational nurses in the workplace. Journal of Nursing Administration.

[b11] Estabrooks CA, Midodzi WK, Cummings GG, Ricker KL, Giovannetti P (2005). Determining the impact of hospital nursing characteristics on 30-day mortality among patients in Alberta acute care hospitals. Nursing Research.

[b12] Fishbein M, Ajzen I (1975). Belief, Attitude, Intention and Behavior: An Introduction to Theory and Research.

[b13] Gregory DM, Way CY, LeFort S, Barrett BJ, Parfrey PS (2007). Predictors of registered nurses’ organizational commitment and intent to stay. Health Care Management Review.

[b14] Griffeth R, Hom P, Gaertner S (2000). A meta-analysis of antecedents and correlates of employee turnover: update, moderator tests, and research implications for the millennium. Journal of Management.

[b15] Hayes LJ, O’Brien-Pallas L, Duffield C, Shamian J, Buchan J, Hughes F, Spence Laschinger HK, North N, Stone PW (2006). Nurse turnover: a literature review. International Journal of Nursing Studies.

[b16] Health Canada (2007). The working conditions of nurses: confronting the challenges. Health Policy Research Bulletin.

[b17] Hinshaw AS, Smeltzer CH, Atwood JR (1987). Innovative retention strategies for nursing staff. The Journal of Nursing Administration.

[b18] Hom PW, Griffeth RW (1995). Employee Turnover.

[b19] Hu J, Herrick C, Hodgin K (2004). Managing the multigenerational nursing team. The Health Care Manager.

[b20] Ingersoll GL, Olsan T, Drew-Cates J, DeVinney BC, Davies J (2002). Nurses’ job satisfaction, organizational commitment, and career intent. The Journal of Nursing Administration.

[b21] Irvine DM, Evans MG (1995). Job satisfaction and turnover among nurses: integrating research findings across studies. Nursing Research.

[b22] Krueger RA (1998). Analyzing and Reporting Focus Group Results.

[b23] Krueger RA, Casey MA (2000). Focus Groups: A Practical Guide for Applied Research.

[b24] Lacey SR, Cox KS, Lorfing KC, Teasly SL, Sexton K (2007). Nursing support, workload, and intent to stay in magnet, magnet-aspiring, and non-magnet hospitals. The Journal of Nursing Administration.

[b25] Larrabee JH, Janney MA, Ostrow CL, Withrow ML, Hobbs GR, Burant C (2003). Predicting registered nurse job satisfaction and intent to leave. The Journal of Nursing Administration.

[b26] Lincoln YS, Guba EG (1985). Naturalistic Inquiry.

[b27] Lu H, While AE, Barriball KL (2005). Job satisfaction among nurses: a literature review. International Journal of Nursing Studies.

[b28] McNeese-Smith D, Crook M (2003). Nursing values and a changing workforce. Journal of Nursing Administration.

[b29] Miles MB, Huberman AM (1994). Qualitative Data Analysis: A Sourcebook of New Methods.

[b30] Mobley WH, Horner SO, Hollingsworth AT (1978). An evaluation of precursors of hospital employee turnover. Journal of Applied Psychology.

[b31] Morgan DL (1997). The Focus Group Kit.

[b32] Mueller CW, Price JL (1990). Economic, psychological, and sociological determinants of voluntary turnover. Journal of Behavioural Economics.

[b33] Needleman J, Buerhaus P, Mattke S, Stewart M, Zelevinsky K (2002). Nurse-staffing levels and the quality of care in hospitals. The New England Journal of Medicine.

[b34] Price JL (2001). Reflections on the determinants of voluntary turnover. International Journal of Manpower.

[b35] Price JL, Mueller CW (1981). A causal model of turnover for nurses. Academy of Management Journal.

[b36] Shader K, Broome M, Broome CD, West ME, Nash M (2001). Factors influencing satisfaction and anticipated turnover for nurses in an Academic Medical Centre. Journal of Nursing Administration.

[b37] Sourdif J (2004). Predictors of nurses’ intent to stay at work in a university health center. Nursing and Health Sciences.

[b38] Tai TWC, Bame SI, Robinson CD (1998). Review of nursing turnover research, 1977-1996. Social Science and Medicine.

[b39] Taunton RL, Boyle DK, Woods CQ, Hansen HD, Bott MJ (1997). Manager leadership and retention of hospital staff nurses. Western Journal of Nursing Research.

[b40] Tett RP, Meyer JP (1993). Job satisfaction, organizational commitment, turnover intention, and turnover: path analysis based on meta-analytic findings. Personnel Psychology.

[b41] Tourangeau AE, Cranley LA (2006). Nursing intention to remain employed: understanding and strengthening determinants. Journal of Advanced Nursing.

[b42] Tourangeau AE, Giovannetti P, Tu J, Wood M (2002). Nursing-related determinants of 30-day mortality for hospitalized patients. Canadian Journal of Nursing Research.

[b43] Tourangeau AE, Doran DM, McGillis Hall L, O’Brien-Pallas LL, Pringle DD, Tu JV, Cranley LA (2007). Impact of hospital nursing care on 30-day mortality for acute medical patients. Journal of Advanced Nursing.

[b44] Wilson-Keates B, Squires M, Widger K, Cranley L, Tourangeau AE (2008). Job satisfaction among a multigenerational nursing workforce. Journal of Nursing Management.

[b45] Yin JC, Yang KP (2002). Nursing turnover in Taiwan: a meta-analysis of related factors. International Journal of Nursing Studies.

[b46] Zemke R, Raines C, Filipczak B (2000). Generations at Work: Managing the Veterans, Boomers, Xers, and Nexters in your Workplace.

[b47] Zeytinoglu IU, Denton M, Davies S, Baumann A, Blythe J, Boos L (2006). Retaining nurses in their employing hospitals and in the profession: effects of job preference, unpaid overtime, importance of earning and stress. Health Policy.

[b48] Zeytinoglu IU, Denton M, Davies S, Baumann A, Blythe J, Boos L (2007). Deteriorated external work environment, heavy workload and nurses’ job satisfaction and turnover intention. Canadian Public Policy.

